# *In Vivo* Experiments with Dental Pulp Stem Cells for Pulp-Dentin Complex Regeneration

**DOI:** 10.1155/2015/409347

**Published:** 2015-11-24

**Authors:** Sunil Kim, Su-Jung Shin, Yunjung Song, Euiseong Kim

**Affiliations:** ^1^Department of Conservative Dentistry, Gangnam Severance Dental Hospital, College of Dentistry, Yonsei University, Seoul 06273, Republic of Korea; ^2^Department of Conservative Dentistry, Hallym Hospital, Hallym University, Anyang 14068, Republic of Korea; ^3^Microscope Center, Department of Conservative Dentistry and Oral Science Research Center, College of Dentistry, Yonsei University, Seoul 03722, Republic of Korea

## Abstract

In recent years, many studies have examined the pulp-dentin complex regeneration with DPSCs. While it is important to perform research on cells, scaffolds, and growth factors, it is also critical to develop animal models for preclinical trials. The development of a reproducible animal model of transplantation is essential for obtaining precise and accurate data* in vivo.* The efficacy of pulp regeneration should be assessed qualitatively and quantitatively using animal models. This review article sought to introduce* in vivo* experiments that have evaluated the potential of dental pulp stem cells for pulp-dentin complex regeneration. According to a review of various researches about DPSCs, the majority of studies have used subcutaneous mouse and dog teeth for animal models. There is no way to know which animal model will reproduce the clinical environment. If an animal model is developed which is easier to use and is useful in more situations than the currently popular models, it will be a substantial aid to studies examining pulp-dentin complex regeneration.

## 1. Introduction

Since Kakehashi et al. reported that bacteria caused pulpitis in their mouse experiment in 1965, endodontic treatment has developed with the goal of the complete removal of bacteria from the root canal [1]. It was believed that, once exposed to bacteria, the dental pulp would fall into a state of irreversible pulpitis that could never be restored to the normal state and that the progression of inflammation would result in necrosis. Therefore, the dental pulp tissues that are damaged due to bacterial invasion or dental trauma should be removed. The conventional root canal treatment focuses on three-dimensional mechanical preparation, disinfection, and the tight sealing of the root canal space to completely eliminate the dental pulp and bacteria in the root canal and prevent reinfection. However, this treatment is only a measure aimed at repair rather than true healing/regeneration. Thus, many studies have been conducted in the field of dental endodontics with the aim of pulp regeneration.

Two approaches have been undertaken for dental pulp-dentin complex regeneration. The first approach is the revascularization procedure. Many case reports have found clinical success following revascularization procedures, for example, no symptoms and no periapical lesions [2–4]. However, the histological observations from animal experiments have revealed that the tissues formed in the root canal do not reflect the regeneration of pulp-dentin but are rather formed of periodontal tissues, such as cementum, periodontal ligament, and bone [5, 6]. The revascularization procedure has its own clinical advantages in the treatment of immature teeth, but it does not result in pulp-dentin complex regeneration in the true sense.

The second approach for regenerating the dental pulp-dentin complex is the transplantation of mesenchymal stem cells into endodontically treated canals. In recent years, various stem cells have been isolated from the oral cavity. In 2000, Gronthos et al. isolated dental pulp stem cells (DPSCs) from human 3rd molars for the first time, and these DPSCs were characterized as highly proliferative cells with self-renewal multidifferentiation properties* in vitro* [7]. The dental pulp is vascularized and characterized by innervated loose connective tissue that contains heterogeneous cell populations [8]. Due to the complexity of pulpal tissues, no universally accepted protocols or cell types are currently available to assess pulp regeneration. However, a consensus exists regarding the importance of neural and vascular reinnervation for successful pulp regeneration [9]. DPSCs that exhibit pluripotent mesenchymal stem cell characteristics can be easily isolated from teeth [10]. Therefore, the strong angiogenic and neurogenic potentials of DPSCs have attracted much attention in the study of pulp regeneration [11–13].

Tissue engineering approaches are based on three central components: the living cells, scaffolds, and growth factors. Various types of stem cells, scaffolds, and growth factors have been researched and reported to function in pulp-dentin complex regeneration. Before applying these candidate substances in clinical trials, their biocompatibilities and treatment efficacies should be evaluated* in vivo* using animal models. In pulp-dentin complex regeneration research, the absence of animal models that adequately reflect pulp injury and pulpitis for investigation of the use of DPSCs has resulted in skipping preclinical testing. However, if the efficacy and safety of a treatment have not been validated in animal experiments, serious side effects can occur [14, 15]. Thus, the development of a reproducible transplantation animal model is essential for obtaining precise and accurate data* in vivo* [16]. The efficacy of pulp regeneration should be assessed qualitatively and quantitatively via the use of animal models.

This review article sought to introduce* in vivo* experiments that have evaluated the possibilities of dental pulp stem cells for pulp-dentin complex regeneration. This paper concentrates on the selection of* in vivo* and DPSC experiments that were identified in the Medline database via PubMed.

## 2. *In Vivo* Engineering in Dental Pulp Regeneration

### 2.1. Ectopic Transplantation versus Orthotopic Transplantation

The use of preclinical animal models is the most informative approach to obtaining clinically relevant data and verifying safety prior to human application. Animal model designs are categorized into ectopic transplantation and orthotopic transplantation. The word “ectopic” is from the Modern Latin from the Greek word* ektopos,* which means away from an original place or abnormal position. The word “orthotopic” is from the Greek word* orthos*, which means normal or usual position.

In pulp-dentin complex regeneration research, the ectopic implantation of stem cell involves transplantation of the cells into the outside of the tooth, for example, into subcutaneous tissue or the renal capsule. Orthotopic implantation involves the transplantation of the cells into the root canal space.  Table 1 summarized current* in vivo* transplantation experiments with dental pulp stem cells.

### 2.2. *In Vivo* DPSCs Experiments: Ectopic Transplantation

It is not easy to apply the various types of cells, growth factors, and scaffolds that can be used controllably in* in vitro* experiments using animals. In early stages,* in vivo* experiments require large numbers of animals and easy access to the points of application. Ectopic transplantation not only has the advantage of easy access but also has the advantages of being rapid and reproducible, requiring minimal labor, being relatively inexpensive, and producing easily quantifiable data [36]. Ectopic implantation is typically chosen for investigations of the characteristics of newly discovered stem cells and studies of the suitability of various scaffolds for dental tissue engineering [37]. Small animals, such as mice or rats, are preferred over large animals, such as monkeys, dogs, and pigs, due to costs, breeding management, and ethical issues.

Mice are the most commonly used species in ectopic transplantation experiments. Mice are relatively inexpensive and easy to handle and involve fewer ethical issues. Mouse anatomy is well known to researchers, and the mouse genome is 99% similar to the human genome [38]. Advances in genetic engineering technology have allowed for the generation of genetically modified mice. Severe combined immune deficiency (SCID) mice are naturally born without an immune system and can serve as models for allogeneic and xenogeneic DPSC transplantations.

Almost all of the ectopic DPSCs transplantation experiments have been performed in mouse subcutaneous tissue. Subcutaneous transplantation is technically easy compared with other sites and is less likely to result in the death of the animal. Many researchers have studied the properties of the mesenchymal stem cells derived from human dental tissue using mouse subcutaneous tissues. Shi et al. obtained dental pulp from impacted 3rd molars and digested tissues in collagenase/dispase to generate single-cell suspensions. Cultured DPSCs were cotransplanted with hydroxyapatite/tricalcium phosphate (HA/TCP) particles into the subcutaneous tissues of immunocompromised mice for 8 weeks. Consequently, donor-derived pulp-dentin-like tissues with distinct odontoblast layers lining the mineralized dentin matrix were generated [17]. Researchers have demonstrated the pulp-dentin complex regeneration capabilities of DPSCs in a variety of environments with mice since the initial* in vivo* and* in vitro* experiments identified the basic characteristics of DPSCs. Okamoto et al. cultured DPSCs with simvastatin for 7 days and transplanted DPSC-HA/TCP mixtures into the dorsal subcutaneous tissues of 10-week-old immunocompromised mice for 8 weeks. The DPSCs pretreated by high-concentration simvastatin formed significantly greater amounts of mineralized tissue than those treated with low concentrations and the controls [18]. Alongi et al. conducted an experiment with mice to examine whether DPSCs could be found in inflamed pulp as well as in normal pulp and if the DPSCs from inflamed pulp would retain their tissue regeneration potential following successful cultivation. These authors reported that DPSCs obtained from inflamed pulp also exhibited tissue regeneration capacities [19]. Huang et al. established an artificial human root canal model in which one end of the canal was opened to permit blood supply and the other end was sealed with MTA [20]. In research in which human root fragments were implanted into immunocompromised mice, DPSC cultures were observed to result in pulp-like tissues with well-established vascularities. This study provides the first evidence that pulp-like tissue can be generated by DPSCs in the empty root canal space and that odontoblast-like cells proliferate from DPSCs to produce dentin-like hard tissue on the dentinal walls. This research is significant in that it used a human root model that created an environment as similar as possible to that of orthotopic implantations to overcome the limitations of ectopic implantation. Additionally, research has been performed to assess the effectiveness of scaffolds and to identify the factors that influence the differentiation of DPSCs into odontoblasts [21–23, 25].

In addition to subcutaneous tissues, renal capsules are used in DPSC research. Yu et al. reported several* in vivo* studies using rat renal capsules [26–28]. The renal subcapsular space is a good site for cell transplantation due to its high graft uptake rate and abundant blood supply [39]. However, renal capsules are not used frequently because they are difficult to access compared with subcutaneous tissues.

### 2.3. *In Vivo* DPSCs Experiments: Orthotopic Transplantation

In dental pulp regeneration research, orthotopic transplantations involve implantation into the teeth, that is, the original anatomical site. When teeth are used in research, they can reflect the actual clinical environment better than ectopic sites such as subcutaneous tissues and renal capsules. However, it is not easy to implant a mixture of DPSCs and scaffolds into animal teeth. This procedure requires particularly advanced skills and techniques when using mice or rats, which have tooth diameters of only 2–5 mm. Therefore, larger animals, such as dogs and pigs, are often preferred over mice for this purpose [14].

Many dental experimental studies have been performed in dogs. Dogs are considered an ideal animal model for dental research due to the similarities in anatomy, growth patterns, and pathophysiologies with humans [40]. Dogs are diphyodonts with deciduous and permanent dentitions. The composition of the permanent dentition is three incisors, one canine, four premolars, and two molars in the maxilla and three incisors, one canine, four premolars, and three molars in the mandible. The beagle is one of the most commonly used dogs because this breed has a well-adjusted temperament and is easy to handle due to its proper size [41].

Almost all research into the orthotopic transplantation of DPSCs has been performed in dogs. Iohara et al. reported various studies about DPSC implantations in dog teeth [29–33]. In 2004, the efficacy of BMP2 on the differentiation of DPSCs into odontoblasts was examined in a canine amputated pulp model. A surgical exposure was made in the canine, and the amputation was performed. The upper incisor pulp was extracted and isolated, and the pulp cells were enzymatically separated. The pellet of the dog pulp cells was cultured and applied to the amputated root canal. Consequently, the transplantation of the BMP2-treated pulp cells into the amputated root canal stimulated reparative dentin formation [29]. Subsequently, Iohara et al. studied several factors that affect pulp regeneration using a surgically amputated canine pulp model. These authors researched the potential utility of a subfraction of a side population of cells, that is, CD31^−^/CD146^−^ and CD105^+^ immunophenotype cells, from dog dental pulp for angiogenesis/vasculogenesis and pulp regeneration in a surgically amputated canine pulp model [30, 31]. These authors demonstrated successful pulp-dentin complex regeneration in canine teeth. The same group also studied the effects of granulocyte colony-stimulating factor and host age on pulp regeneration [32, 33].

In addition to Iohara, other researchers have conducted research on pulp regeneration in dogs. Zhu et al. investigated the pulp regeneration potential of DPSCs and platelet-rich plasma (PRP) using the surgically amputated canine pulp model established by the Iohara group [34]. Contrary to the expectation that better results would be observed in a DPSC and PRP group than in a blood clot group, histologic examinations did not indicate any differences between the two groups. These findings opposed those from previous* in vitro* research that predicted that DPSCs plus PRP would play an important role in pulp-dentin complex regeneration because PRP contains many growth factors, and treatment with the appropriate concentration of PRP has been shown to enhance the proliferation and mineralization differentiation of DPSCs [24], which indicates that the results obtained in the laboratory may not lead to results in preclinical animal studies.

Several studies have employed mini pigs. Kodonas et al. demonstrated the regenerative capacity of swine DPSCs seeded in organic and synthetic scaffolds and implanted into the jaw bones of mini pigs [35]. These authors established a root implant model. The root implants were manufactured using the middle part of the roots of freshly extracted swine incisors, and the root canals were filled with scaffolds containing DPSCs and then implanted into the fresh postextraction sockets. This method is not truly orthotopic transplantation. However, it can be used as a valuable animal model for DPSC research in that it attempts to reproduce the clinical environment as much as possible.

### 2.4. Other Orthotopic Transplantation Models

Although not truly DPSC-based research, other efforts have been made to create an animal model to identify the elements that influence pulp regeneration [42, 43]. Torabinejad performed radiographic and histologic examinations with ferret cuspid teeth over 36–133 days after birth [43]. This author reported that ferret cuspid teeth exhibit anatomical, physiologic, histologic, and pathologic characteristics that are similar to those of human teeth. The ferret has four single-root teeth that are easily accessible for the performance of endodontic procedures and easy to evaluate radiographically and histologically. Torabinejad's model holds some significance as an animal model because ferrets are easier to handle and are cheaper than dogs and present fewer ethical issues than dogs, which are popular pets.

Other researchers have created orthotopic animal models with small animals instead of large animals, such as dogs, pigs, and ferrets. Simon et al. evaluate mice as an* in vivo* model for the study of pulpal healing in response to direct pulp capping [42]. These authors proposed the possibilities of orthotopic transplantation research with mouse teeth in preliminary studies despite the difficult accessibility and the small sizes of the teeth. Despite their many advantages as experiment animals, small animals, such as rodents, have been considered to be inappropriate for orthotopic transplantation animal models due to their small teeth [14, 43]. However, it became possible to apply DPSCs to the teeth of small animals and to perform histological and radiological observations of those teeth because of the use of microscopes, the developments in experimental instruments, and the introduction of scaffold-free approaches [42, 44]. Additional research on the development of rodent animal models will aid the development of new orthotopic models.

## 3. Conclusion 

Recently, many studies have approached the pulp-dentin complex regeneration with DPSCs. While it is important to perform research on cells, scaffolds, and growth factors, it is also critical to develop animal models for preclinical trials. Although no human studies have yet been performed, research has been conducted with various types of animals and produced promising results.

According to a review of various studies of DPSCs, the majority of studies have used mouse subcutaneous tissues and dog teeth for animal models. There is no way to know which animal model better reproduces the clinical environment, but the results of an orthotopic transplantation experiment will be more reliable than those of ectopic transplantation experiments. The problem is that orthotopic transplantation research lags behind ectopic transplantation research in both quantity and quality. Sufficient orthotopic transplantation research should be conducted before clinical trials are initiated. If an animal model is developed which is easier to use and more versatile than the currently popular models (e.g., the canine pulp model), that model will be of substantial benefit to research on pulp-dentin complex regeneration.

## Figures and Tables

**Table 1:** Description of *in vivo* experiments with the application of DPSCs for pulp-dentin complex regeneration.

Author	Animal	Ectopic/orthotopic	Source of stem cell
Shi et al., 2005 [17]	IC mice	Ectopic (subcutaneous)	hDPSCs, SHED, hPDLSCs
Okamoto et al., 2009 [18]	IC mice	Ectopic (subcutaneous)	hDPSCs
Alongi et al., 2010 [19]	IC mice	Ectopic (subcutaneous)	hDPSCs
Huang et al., 2010 [20]	IC mice	Ectopic (subcutaneous)	hDPSCs, SCAP
Wang et al., 2010 [21]	IC mice	Ectopic (subcutaneous)	hDPSCs
Galler et al., 2011 [22]	IC mice	Ectopic (subcutaneous)	hDPSCs, SHED
Lee et al., 2011 [23]	IC mice	Ectopic (subcutaneous)	hDPSCs
Choung et al., 2013 [25]	IC mice	Ectopic (subcutaneous)	hDPSCs
Yu et al., 2006 [26]	Rats	Ectopic (renal capsule)	hDPSCs
Yu et al., 2007 [27]	Rats	Ectopic (renal capsule)	hDPSCs, BMSSCs
Yu et al., 2008 [28]	Rats	Ectopic (renal capsule)	hDPSCs
Iohara et al., 2004 [29]	Dogs	Orthotopic (canine)	Porcine pulp cells
Iohara et al., 2009 [30]	Dogs	Orthotopic (canine)	SP cells from canine pulp
Iohara et al., 2011 [31]	Dogs	Orthotopic (incisor)	SP cells from canine pulp
Iohara et al., 2013 [32]	Dogs	Orthotopic (incisor)	MDPSCs
Iohara et al., 2014 [33]	Dogs	Orthotopic (incisor)	MDPSCs
Zhu et al., 2012 [34]	Dogs	Orthotopic (premolar)	cDPSCs
Kodonas et al., 2012 [35]	Mini pigs	Orthotopic (incisor root, jaw bone)	sDPSCs

IC, immunocompromised; hDPSCs, human dental pulp stem cells; SHED, stem cells from human exfoliated deciduous teeth; hPDLSCs, human periodontal ligament stem cells; SCAP, stem cells from apical papilla; BMSSCs, bone marrow stromal stem cells; SP, side population; MDPSCs, DPSC subfraction based on their migratory response to granulocyte-colony stimulating factor; cDPSCs, canine dental pulp stem cells; sDPSCs, swine dental pulp stem cells.
